# Compact Microfluidic Platform with LED Light-Actuated Valves for Enzyme-Linked Immunosorbent Assay Automation

**DOI:** 10.3390/bios12050280

**Published:** 2022-04-27

**Authors:** Mireia Burdó-Masferrer, María Díaz-González, Ana Sanchis, Álvaro Calleja, María-Pilar Marco, César Fernández-Sánchez, Antonio Baldi

**Affiliations:** 1 Institut de Microelectronica de Barcelona, IMB-CNM (CSIC), Campus UAB, 08193 Bellaterra, Spain; mireia.burdo@gmail.com (M.B.-M.); maria.diaz.cnm@gmail.com (M.D.-G.); alvaro.calleja@imb-cnm.csic.es (Á.C.); cesar.fernandez@imb-cnm.csic.es (C.F.-S.); 2 Institut de Química Avançada de Catalunya (IQAC-CSIC), 08034 Barcelona, Spain; asvillariz@gmail.com (A.S.); pilar.marco@cid.csic.es (M.-P.M.); 3 CIBER de Bioingeniería, Biomateriales y Nanomedicina (CIBER-BBN), Jordi Girona 18-26, 08034 Barcelona, Spain

**Keywords:** lab-on-a-chip, lab-on-a-foil, wax valve, microfluidic ELISA

## Abstract

Lab-on-a-chip devices incorporating valves and pumps can perform complex assays involving multiple reagents. However, the instruments used to drive these chips are complex and bulky. In this article, a new wax valve design that uses light from a light emitting diode (LED) for both opening and closing is reported. The valves and a pumping chamber are integrated in lab-on-a-foil chips that can be fabricated at low cost using rapid prototyping techniques. A chip for the implementation of enzyme-linked immunosorbent assays (ELISA) is designed. A porous nitrocellulose material is used for the immobilization of capture antibodies in the microchannel. A compact generic instrument with an array of 64 LEDs, a linear actuator to drive the pumping chamber, and absorbance detection for a colorimetric readout of the assay is also presented. Characterization of all the components and functionalities of the platform and the designed chip demonstrate their potential for assay automation.

## 1. Introduction

In recent years, lab-on-a-chip devices have become an important tool for biosensing and diagnostic applications [1,2,3]. However, there is still a major challenge to overcome, which is the integration of actuators (e.g., microvalves and micropumps) that can be driven with a compact instrument, especially when these devices are intended to carry out analytical assays that involve multiple reagents. The pumping of each reagent with a dedicated linear actuator has been used to achieve their sequential release, as required by the assay protocol [4,5]. This approach allows good flow-rate control, but limits the number of reagents that can be managed to the number of actuators contained within the instrument.

Another approach is the use of valves to control the sequential reagent release. Many different valving mechanisms have been proposed for their integration in microfluidic chips [6]. Membrane valves have been a frequent choice [7,8,9]. However, they require an external pneumatic or mechanical actuator per each independent valve, which hinders the development of compact instruments when a large number of valves are required. A more suitable alternative for larger scale integration is the use of barrier-type microvalves [10,11,12,13,14,15,16,17,18,19,20]. The operation of these valves relies on the phase-change characteristics of the barrier materials (e.g., paraffin) to displace or deform them, allowing the passage or blockage of fluids. The softening or melting of the material has been achieved by electrical or optical means. Electrical heaters [10,11,12,13,14,15,16] require conductive tracks and electrical connections to an external current source, which complicates the chip fabrication and its interface with the instrument. Laser heating of the barrier material [17,18,19,20,21] avoids electrical connections, but requires precise positioning and focusing systems that are difficult to miniaturize.

Here, a new platform that uses light emitting diode (LED) light for valve control is explored. The alignment of the valves in the chip with the LEDs on the instrument is straightforward. The control of multiple reagents with a single pumping component is demonstrated. The platform also includes absorbance measurements in order to implement microfluidic enzyme-linked immunosorbent assays (ELISA). All the components of the chip are integrated using a lab-on-a-foil structure that can be fabricated at a low cost with rapid prototyping techniques [22,23]. A chip for the detection of tumor necrosis factor alpha (TNFα), which is a cytokine molecule involved in many inflammatory processes [24], is presented.

## 2. Materials and Methods

### 2.1. LED Light-Actuated Wax Valves

**Figure 1:**
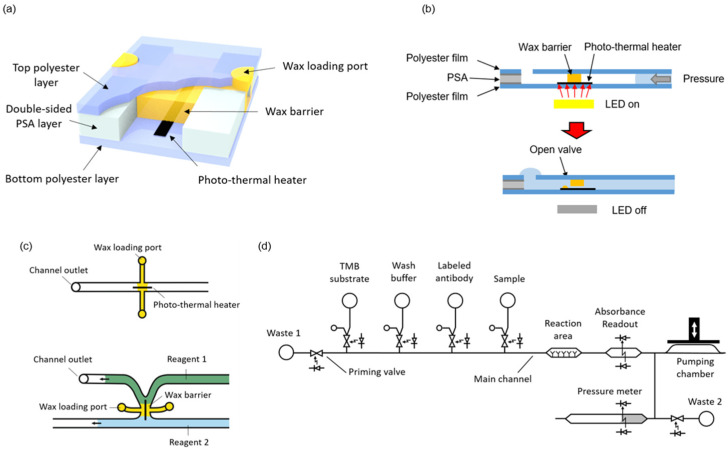
(**a**) A 3D illustration of the valve structure; (**b**) Cross-sectional representation of the valve opening; (**c**) Two different configurations of the wax valve; (**d**) Complete fluidic schematic of the ELISA chip.

Microvalves were fabricated by integrating wax barriers and heaters into microfluidic chips (Figure 1a). The heaters are based on the photothermal effect, which is a non-radiative conversion of absorbed electromagnetic energy leading to a temperature increase [25]. The chips consist of one patterned double-sided pressure sensitive adhesive (PSA) layer sandwiched between two transparent polyester films. The photo-thermal heaters, made of black toner, are printed onto the bottom film and positioned to lie across the wax barriers. Black toner contains carbon black and iron oxide, which are efficient light-to-heat conversion materials [26]. White light pulses from LEDs placed on the instrument are used to transfer energy and raise the temperature of the heaters. Simultaneous to the light pulse, a pressure difference is applied across the valve. When the wax in close contact with the activated heater melts, it is rapidly ejected out of the barrier. In this way, a small tunnel is generated just on top of the heater, and the liquids (and gases) can flow through the valve (Figure 1b). In order to close the valve, the heater is activated again by a light pulse, but with no pressure difference applied across the barrier. In these conditions, the melted wax refills the tunnel and, once solidified, blocks the passage of fluids.

As illustrated in Figure 1c, the microvalves can be integrated either in a microchannel or in the junction between two microchannels. The latter configuration is convenient to avoid dead volumes and air trapping during the priming of the system. In the present design, the microchannels were 0.5 mm wide and 120 µm high, and the heater line was 100 µm wide. No negative effect in the characteristics of enzyme-linked assays has been described in previous works using wax for valves or as structural microfluidic material in contact with the sample or reagents [17,27].

The complete system to perform automated immunoassay requires the integration of some additional structures besides the valves, namely a pumping chamber, reservoirs for the reagents, a reaction area, and absorbance measurement areas. Figure 1d shows a fluidic schematic of the system. These additional components are presented in the following sections.

### 2.2. Pumping Chamber

**Figure 2:**
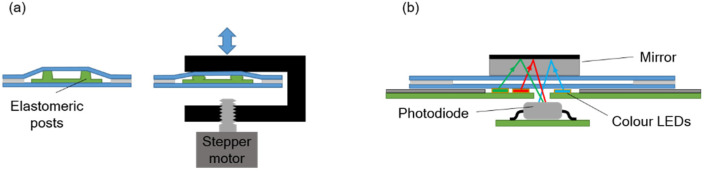
Schematic illustration of (**a**) the pumping system, and (**b**) the absorbance measurement system.

The pressure required to move the fluids inside the chip is provided by a pumping chamber, which is actuated with an external stepper-motor linear actuator integrated in the instrument. An array of polydimethylsiloxane (PDMS) elastomeric posts inside the chamber enables expansion of its internal volume when the actuator is moved upwards, so that both positive and negative pressure can be generated (Figure 2a).

A single pump, in combination with the valves, is enough to manage all the reagents by aspirating them from the end of the main channel. The pump is filled with a solution that is used to prime the main channel at the beginning of the assay.

### 2.3. Reagent Reservoirs

The reservoirs consist of simple cylindrical cuvettes attached on top of inlet orifices. Each inlet is connected to a valve with a short channel that ends in a vent orifice. When the cuvette is filled with the reagent, the channel fills, owing to the small hydrostatic pressure present at the bottom of the reservoir. The vent orifice functions as a burst valve and stops the flow of liquid once the channel is completely filled. In this way, the presence of trapped air that could be injected into the main channel is avoided.

### 2.4. Reaction Area

The reaction area is located in the main channel and contains the immobilized capture antibodies (the so-called solid phase of the immunoassay). Both the capture of the target molecule and the accumulation of enzymatic products at the end of the immunoassay are favored when a large surface to volume ratio structure is used as the solid phase [28]. The filling of a microchannel with packed beads has been often proposed as a way to provide a large surface for antibody immobilization. However, the porous-like structure of packed beads generates a large fluidic resistance and the trapping of bubbles, which makes it difficult to obtain a stable flow of reagents through the channels [29].

In the present design, a nitrocellulose strip was used for the immobilization of the capture antibodies. The strip was 60 µm high, 500 µm wide, and 2000 µm long. The main channel was 120 µm high and 500 µm wide. Therefore, the nitrocellulose occupied only half of the channel height. Nitrocellulose has a high surface to volume ratio because of its porous structure. By leaving half of the channel cross-section free for the reagents to flow, the high fluidic resistance and bubble-trapping problems were avoided.

The immunoassay readout is based on the absorbance detection of blue colored products from the reaction of 3,3′,5,5′-Tetramethylbenzidine (TMB) by hydrogen peroxide catalyzed using the horseradish peroxidase (HRP) enzymatic label. To that end, the generated enzymatic products are flown through the absorbance measurement area located in the main channel 5 mm downstream from the reaction area (Figure 1d).

### 2.5. Absorbance Measurement for Assay Read-Out, Reagent Identification, and Pressure Measurement

The absorbance measurement is carried out with an LED-photodiode scheme as shown in Figure 2b. A mirror attached to the top chip surface reflects the light from the LED and redirects it back towards the photodiode inside the instrument. Three LEDs emitting blue, green, and red light are used in order to detect absorbance at different wavelengths. Their emitting spectra are centered at 470 nm, 530 nm, and 629 nm, correspondingly.

For the assay readout, the red LED was used, since it emits close to the maximum absorbance wavelength of the blue-colored oxidized TMB species (652 nm) [30].

The absorbance measurement can also be used to detect the passage of the reagents through the main channel, which allows for the monitoring of the correct progress of the assay protocol. To that end, different color dyes were added to the reagent solutions (except to the TMB solution).

Finally, the photodiode signal is also applied for the detection of pressure changes inside the microchannels. In this case, a dead-end channel is partially filled with a dark solution and the liquid-air interphase is positioned on top of the absorbance measurement LEDs. Pressure variations expand or contract the air trapped in the dead-end channel, displacing the liquid-air interphase, and in turn, producing changes in the absorbance signal.

### 2.6. Instrument Design

A generic instrument with an array of 8 × 8 white power LEDs for valve control, 14 LED-photodiode measurement points, and one linear actuator was developed. This generic instrument allows for different chip designs to be tested, as long as the integrated valves, the absorbance measurement areas, and the pumping chamber positions in the chip match with the corresponding components available on the instrument. White LEDs in the array are spaced 1 cm apart. The valves in the chips have to be positioned directly on top of an LED. LED emission is produced in a square area of 1.5 × 1.5 mm^2^. The area of the valve that has to be irradiated during actuation is the intersection of the heater and the wax barrier footprints, which fits in a 0.4 × 0.4 mm^2^ area when all the orientations of the valve are taken into account. This yields a misalignment tolerance of 0.55 mm in any direction. Four posts on the surface of the instrument that match the position of four circular orifices on the chip are used to guarantee a sufficiently good alignment of the valves with the LEDs.

**Figure A1:**
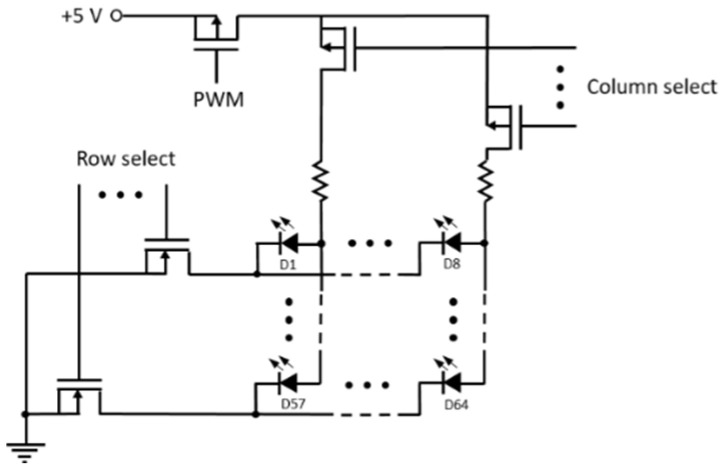
Circuit for LED row-column addressing.

The instrument basic functions are controlled with an Arduino board. Row-column addressing is used for the activation of a particular LED, and pulse width modulation if used for the regulation of the power delivered during the light pulse (Figure A1).

A program with the list of commands to be executed for the implementation of the assay protocol is written and sequentially sent to the Arduino board from a LabView interface in a PC.

### 2.7. Microfluidic Chip Materials

Microcrystalline wax (Ibercer 2616, melting point of 81 °C) was kindly provided by Iberceras Specialities S.L.U. (Madrid, Spain). We purchased 100 µm-thick polyester transparency A4 sheets from APLI S.L. (Alaquàs, Spain). Pressure-sensitive-adhesive films (PSA) (ARcare^®^ 8939, 120 µm total thickness) were obtained from Adhesives Research Inc. (Glen Rock, PA, USA). Poly(methyl methacrylate) (PMMA) was acquired from Servicio Estación (Barcelona, Spain). Unbacked 120 µm-thick nitrocellulose was obtained from GE Healthcare Life Sciences (Piscataway, NJ, USA). Polydimethylsiloxane (PDMS Sylgard 184 kit, which includes the Sylgard 184 silicone elastomer base and the Sylgard 184 silicone elastomer-curing agent) was purchased from Sigma Aldrich (St. Louis, MO, USA). For the immunoassay, the capture (anti-human) and detector antibodies (anti-human-HRP), along with the analyte (TNFα), were obtained from Sinobiological Inc. (Beijing, China). The buffer reagents (Phosphate Buffered Saline (PBS), Tween 20, Bovine Serum Albumin (BSA)), the dyes (Tartrazine, Erioglaucine and Allura red), the streptavidine-HRP, as well as the substrate (3,3′,5,5′-Tetramethylbenzidine (TMB)) were obtained from Sigma Aldrich (USA). Sodium Hydrogen Carbonate (NaHCO_3_) was purchased from Panreac Química (Barcelona, Spain).

### 2.8. Microfluidic Chip Fabrication

The fabrication of the microfluidic chips consists of four main stages. First is the preparation of the different parts, namely the polymer layers, the reservoir cuvettes, the PDMS post array, and the antibody functionalized nitrocellulose strip. Second is the assembly of these parts. Third is the formation of the wax barriers, and fourth is the loading of the pump and the reservoirs with the different buffers and reagents.

The chip was designed with Corel Draw Suite 2018. Toner heaters were printed with a laser printer (Xerox B210) onto the bottom polyester layer. Afterwards, all the polymer films were cut using a laser cutter (Epilog mini 24). Then, the films were washed in a soap and water solution (1 mL soap in 50 mL deionized water) while gently rubbing with a soft sponge, and finally rinsed with deionized water.

The nitrocellulose was compressed in a manual press under a pressure of 4.8 MPa for 2 min in order to reduce its thickness. The final thickness was 60 ± 10 µm. Subsequently, it was cut in 2 × 0.5 mm strips with a cutter plotter (Roland CAMM-1 Servo Cutter). Then, the nitrocellulose was functionalized by drop-casting a 0.2 mg/mL solution of capture antibody and allowing it to dry for 15 min at room temperature. After that, the nitrocellulose was rinsed three times by pipetting a drop of 10 µL of washing buffer (Phosphate Buffered Saline with 0.05% Tween 20 (PBST 0.05%)) on top of each strip and aspirating the buffer in order to remove any excess antibody. Next, the structures were immersed in a blocking solution containing 1% BSA for 15 min at room temperature. Finally, the structures were dried at room temperature for at least 10 min.

The PDMS array of 8 posts was fabricated by molding in a two-layer, laser-cut PMMA mold. The height of the posts and thickness of the base plate was 1.76 mm and 0.33 mm, correspondingly.

For the chip assembly, the bottom polyester and the double-side PSA films were aligned and pressed together with a rubber roller until all microchannel perimeters where completely adhered. Then, the functionalized nitrocellulose strip and the PDMS elastomeric post array were placed in their corresponding positions on the chip. Finally, the top polyester film was aligned and pressed against the other two with the rubber roller. A base with posts that matched round orifices on the films was used for the alignment during assembly. The same orifices were used later on for the alignment of the chips with the instrument.

Cuvettes for the reservoirs were cut out of a 5 mm-thick PMMA plate with the laser cutter. The inner radius was 5 mm, yielding an internal volume of 100 µL. The cuvettes were fixed at their positions on the chip surface with laser-cut double-side PSA rings having the same footprint.

**Figure 3:**
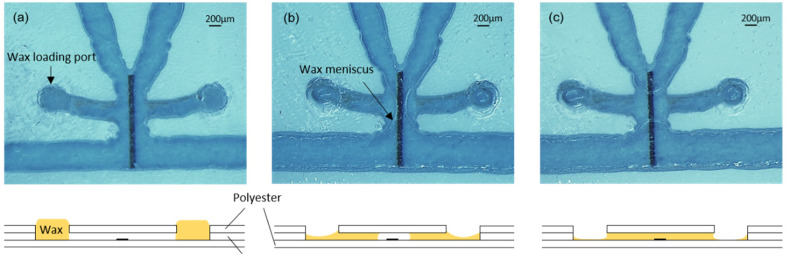
(**a**) Valve with wax-loading port full of wax; (**b**) Penetration of the wax inside the channel by capillarity; (**c**) Formation of the wax barrier on top of the heater. Bottom drawings are cross-sectional illustrations of the process.

For the formation of valves, the chips were placed on a hotplate at 45 °C. A polyester template with orifices matching the filling port orifices was aligned and fixed to the surface of the chip with tape. Then, a small quantity of wax, which had been softened by preheating it at 45 °C, was spread on the template surface and introduced into the filling port orifices with a plastic blade (similar to the screen printing process). The template was carefully detached from the chip surface, and the wax remained inside the filling port orifices (Figure 3a). After that, the hotplate temperature was increased to 90 °C to completely melt the wax and allow it to flow by capillarity into the short lateral microchannels connecting the filling port orifices with the heater areas (Figure 3b). In each valve, the wax from both lateral microchannels advanced by capillarity towards the heater and, once united, created ca. 500 µm long barriers, as shown in Figure 3c. During this step, the reaction area was always kept out of the hotplate in order to avoid the denaturalization of the capture antibodies. Finally, the chip was removed from the hotplate and the wax inside the channels immediately solidified.

The mirrors were fabricated by depositing 1 µm-thick aluminum on a 0.5 mm-thick Pyrex wafer, which was subsequently diced into 5 × 5 mm chips. The deposition of the aluminum was performed by sputtering in the IMB-CNM cleanroom. Two mirrors were positioned on the two absorbance measurement areas used. A 1 mm-thick glass was added underneath each mirror to reach a total separation of 1.5 mm from the surface of the chip to the reflecting surface, which is required to maximize the light received by the photodiode. The mirrors were held in place with adhesive tape.

The pumping chamber was filled with a dyed PBST-BSA 1% solution through a loading channel and orifice. A vent orifice next to the pressure sensing channel was used to remove the air during pump loading and to introduce the dark solution (mixture of green, red, and blue dye solutions) used for pressure measurement. The loading and vent orifices were subsequently sealed with tape.

### 2.9. Instrument Materials and Fabrication

The plastic parts of the instrument were designed with FreeCAD and fabricated with an EOS P100 Formiga 3D printer using PA2200 polyimide. The linear actuator was built with a stepper motor (28BYJ-48) connected to a stainless-steel threaded shaft and a M8 nut glued to a c-shaped 3D-printed part in order to convert rotating movement to linear actuation. An Arduino Mega 2560 board, a stepper motor driver (ULN2003A), white power LEDs (LXZ2-6570), blue LEDs (LXZ1-PB01), red LEDs (L1CU-RED1000000000), green LEDs (LXZ1-PM01), photodetectors (OPT101), p-MOS transistors (TSM2323CX), and n-MOS transistors (TSM2314CX) where mounted on printed circuit boards developed and fabricated in-house.

### 2.10. Immunoassay Protocol

The immunoassay is based on a sandwich procedure, where horseradish peroxidase (HRP) is used as the enzymatic label and 3,3′,5,5′-Tetramethylbenzidine (TMB) as the substrate mediator. Blocking and rinsing buffers are PBST-BSA 1%. Both TNFα and the detector antibody are diluted in a PBST solution containing 1% BSA. The blocking buffer is dyed red (3 mg/mL allura red), the detector antibody is dyed blue (0.8 mg/mL erioglaucine), the TNFα test solution is dyed green (0.16 mg/mL erioglaucine and 1.6 mg/mL tartrazine), and the rinsing buffer is dyed yellow (2 mg/mL tartrazine). No dyes are used for TMB to avoid interference with the absorbance measurement of the enzymatic reaction products.

Once the chip is placed on the instrument, the reagents and the sample are pipetted inside the reservoirs, which produces the spontaneous filling of the reservoir channels. Then, a positive pressure of 10 kPa is applied, and the priming valve in the main channel is opened, resulting in the filling of the main channel with the blocking solution (PBST-BSA 1%) contained in the pumping chamber. The linear actuator is slowly moved to the lowest position and the excess solution in the pumping chamber flows to a waste reservoir located at the end of the main channel. After 30 min, the priming valve is closed and the sample valve is opened. The sample solution containing TNFα is incubated with the nitrocellulose strip in the reaction area by applying a constant flow of 3 µL/min for 15 min. Afterwards, the sample valve is closed and the antibody valve is opened. A 5 min incubation with the anti-human TNFα detector antibody is carried out at 3.6 µL/min. Subsequently, the reaction area is washed with 40 µL of rinsing solution, and the main channel is filled with the TMB substrate at high speed (30 µL/min) by opening and closing the corresponding valves. After 5 min of incubation at zero flow rate, the accumulated blue-colored products are slowly displaced to the detection area with a constant flow of 3 µL/min, while the absorbance signal is recorded. The pumping chamber has to be emptied during the assay because its internal volume is not large enough to aspirate all the liquids involved in the assay in a single stroke. This is carried out through the waste valve between the detector antibody incubation and the washing steps.

## 3. Results and Discussion

**Figure 4:**
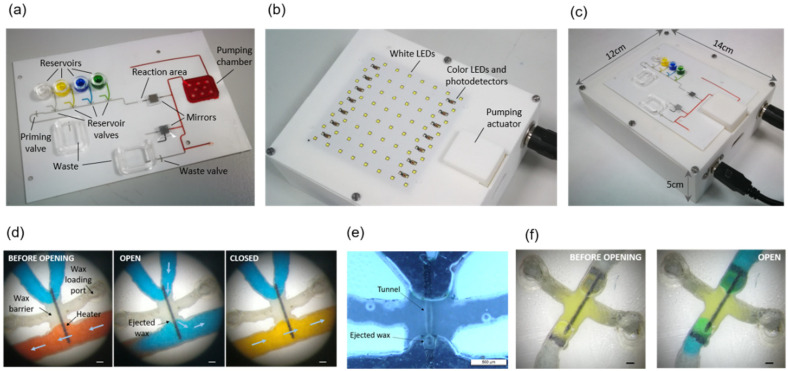
(**a**) Chip; (**b**) Instrument; (**c**) Chip on the instrument; (**d**) Optical microscope images before and after opening and closing a reservoir valve. Arrows indicate the direction of the flow (a white piece of paper was interposed between the valve and the LED during image captures for improved visualization). Scale bars are 200 µm; (**e**) Black field optical microscope image of an open reservoir valve. The liquid has been removed to enhance the visualization of the tunnel. Scale bars are 500 µm; (**f**) Priming valve before and after opening (the LED outline can be seen underneath the valve through the transparent polyester films). Scale bars are 200 µm.

A chip loaded with the reagents and the assembled instrument are shown in Figure 4a,b, respectively. The chip is positioned on the instrument by inserting the alignment posts in the four orifices at its corners (Figure 4c). In this way, each valve in the chip is accurately positioned on top of an LED on the instrument, whereas the chip pumping chamber is positioned under the linear actuator plate.

The sample reservoir is filled with the TNFα sample solution, which is dyed green for easy identification during the immunoassay. The other reservoirs contain the enzyme labelled antibody solution (dyed blue), the washing solution (dyed yellow), and the TMB solution (no dye added). The pumping chamber contains red-dyed blocking solution.

### 3.1. Valves Characterization

Figure 4d shows optical microscope images of the valve before opening, after opening, and after closing. The valve is fully primed before opening, so that no air bubbles are injected upon opening and releasing the blue-dyed solution to the main channel. After closing, a new yellow solution is flown through the main channel and, thanks to the absence of dead volume, the blue solution is rapidly and completely washed out. This is of particular importance when the antibody solution is washed before TMB incubation, since any remains of the enzyme-labelled antibody would produce an unspecific signal during the immunoassay readout.

**Figure A2:**
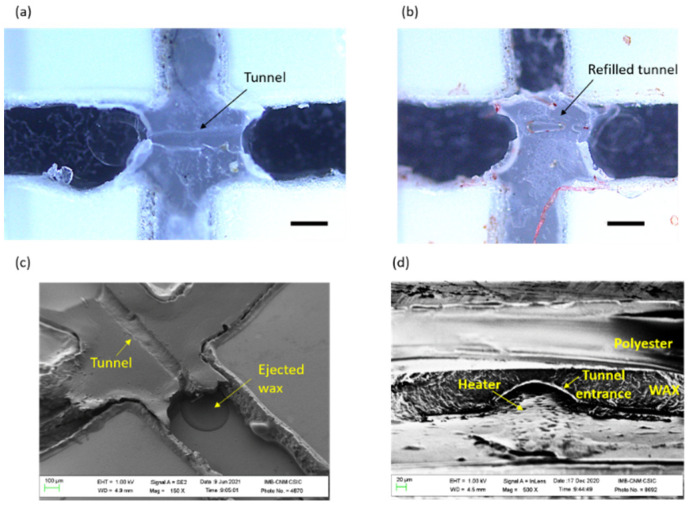
Microscope images of valves with the bottom polyester layer removed (**a**) after opening, and (**b**) after closing. Scale bars are 250 µm; (**c**) SEM image of an open valve with the bottom polyester layer removed; (**d**) SEM image of an open valve from the tunnel entrance side.

A black field image of an open valve after liquid removal allows for observing the shape of the tunnel formed across the wax barrier located on top of the photo-thermal heater (Figure 4e). Further details of the tunnel shape are shown in scanning electron microscope (SEM) images of a disassembled valve in Figure A2. These images show that the wax ejected during tunnel formation attaches to the microchannel floor and ceiling next to the tunnel exit. We have not observed wax traces at any other point downstream of the valves. Figure 4f shows the opening and the shape of the tunnel of a priming valve. In this case, the air at both sides of the valve is pushed out through the waste outlet during the main channel priming.

The valves were both opened and closed with a 100 ms pulse at 80% pulse width modulation (PWM). The pressure applied during opening was 10 kPa. Based on the pulse duration, the modulation value, and the current flowing through the LED (which was estimated by measuring the voltage drop at the series resistor), the estimated energy consumption for both opening and closing is 0.8 joules. These characteristics are similar to previously reported electrically controlled wax valves [16] in terms of response time, but with an order of magnitude increase in consumption. On the other hand, the present light-actuated valves have the advantage of not requiring electrical connections, and therefore, the interface with the instrument is simpler and more reliable. Unlike laser-actuated wax valves used in centrifugal microfluidics [17,20,21], where the whole wax barrier is melted and displaced, with the present valves, only a small tunnel in the wax barrier is opened, which allows resealing them afterwards, and even reopening them, if necessary. Multiple opening and closing was also achieved with magnetically driven wax in [20], but at the expenses of including magnet positioning systems in the instrument.

### 3.2. Pump Characterization

**Figure 5:**
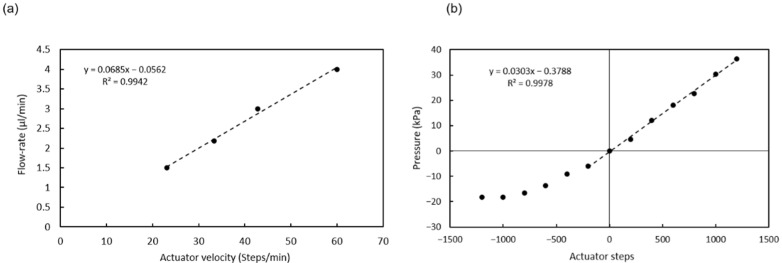
(**a**) Calibration of the flow rate vs. actuator velocity; (**b**) Calibration of the pressure vs. actuator displacement (all valves closed). Dashed lines are guides to the eye.

**Figure A3:**
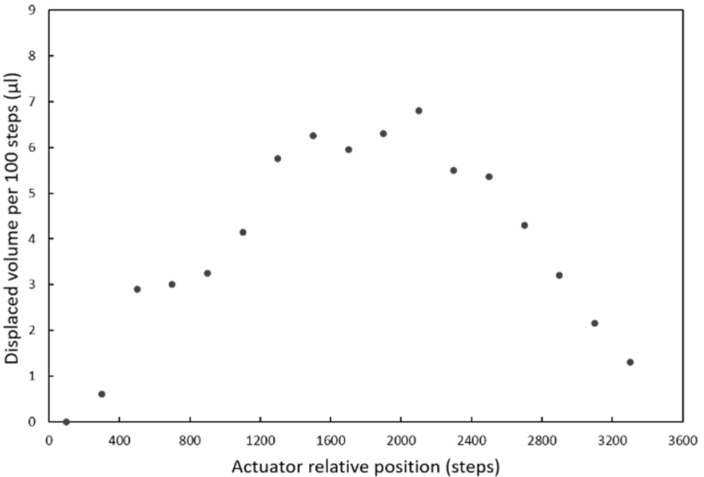
Dependence of the volume displaced by the pumping chamber on the relative position of the actuator.

The linear actuator used to actuate the pumping chamber is based on a 2042 steps/turn stepper motor that when combined with the standard M8 thread in the shaft and nut, yields a 0.61 µm displacement per step. The actuator plate movement compresses the chamber and generates flow or a pressure increase, depending on whether there is an open valve or not. Figure 5a shows the flow rates generated for different actuator velocities when a valve is opened and the liquid can enter or exit the microchannel. Since the 3D-printed actuator plate is not completely rigid, the relation between liquid displacement and actuator movement has some dependency on the actuator’s relative position (Figure A3). In order to minimize this dependency, the pump was used in a short range of steps (1600) where the flow rate was the most stable.

When no valve is open, the actuator movement produces changes in pressure, as shown in Figure 5b. The pressure generated is positive for a downward displacement of the actuator and negative for an upwards displacement of the actuator and the consequent expansion of the PDMS post array inside the chamber. The negative pressure generated is limited by the elasticity of the PDMS posts. The pressure used to open the valves (10 kPa) can be generated with a movement of 400 steps.

### 3.3. Absorbance Measurement Characterization

**Figure 6:**
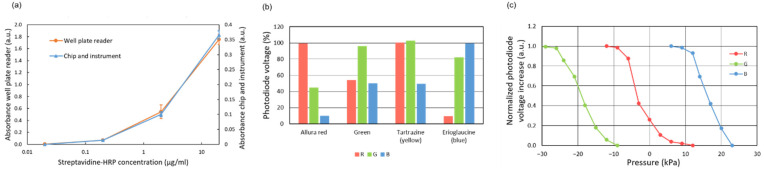
(**a**) Absorbance measurement for oxidized TMB. Comparative with a plate reader; (**b**) Photodiode output voltage signal for different dye solutions (and different activated LEDs) in relation to the values obtained for deionized water. The concentration of dyes is the same used for the reagents; (**c**) Calibration of the pressure measurement based on the compression of air in a dead-end channel.

The sensitivity of the absorbance measurement system was compared to that of a commercially available spectrophotometric plate reader. To that end, 90 µL of fresh TMB solution was incubated for 5 min in wells of a 96-well plate functionalized with streptavidin-HRP conjugate. The well functionalization was carried out by adding 90 µL of solutions of streptavidin-HRP conjugate at different concentrations, in the range of 0.02 to 20 µg/mL, and incubating them for 1.5 h. The partially oxidized TMB was then injected into the main channel. The photodiode signal was compared to that of non-oxidized TMB, and the absorbance increase was calculated with the equation:

A = −log (V_non-ox_/V_ox_) (1)

where V_non-ox_ and V_ox_ are the output voltages of the photodiode amplifier circuit (proportional to the received light power) for the non-oxidized TMB and oxidized TMB solutions, correspondingly. The curves obtained for these absorbance measurements are plotted in Figure 6a. Even though the microchannel measurement involves a much shorter optical path, the minimum concentration detected in the chip was the same as the one detected in the wells by the plate reader (the wells were filled with 90 µL of solution).

The identification of different dyed reagents was also characterized (Figure 6b). Different dye solutions could be clearly distinguished by the pattern of absorbance signals obtained with the three color LEDs. These patterns show the maximum transmission of light for the LEDs with the same color as the dye being identified, as expected.

Finally, the pressure measurement methodology based on absorbance measurements was also characterized. The displacement of the air-liquid meniscus in a dead-end channel was measured by applying different pressure values with the pump while recording the output signal of the photodiode underneath the meniscus. The displacement of the meniscus was also measured using a ruler in an additional dead-end channel to estimate the applied pressure. These measurements are based on the principle that the decrease in volume in a channel upon air compression is directly proportional to the increase in pressure for an ideal gas at constant temperature. The resulting calibration curves are shown in Figure 6c. The response is not linear and does not allow a precise measurement of the absolute pressure. However, the photodiode signal can be used for detecting pressure changes and monitoring the proper functioning of the valves and the pump.

### 3.4. Immunoassay Characterization

**Figure 7:**
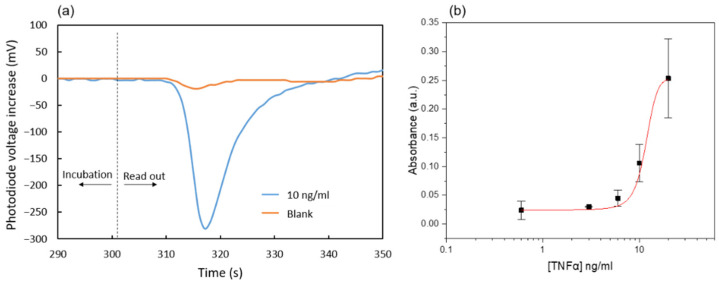
(**a**) Photodiode signals for two TNFα concentrations; (**b**) Absorbance response to different TNFα concentrations. Each data point is the average of three measurements obtained from three separate chips. The mean absorbance value of the blanks has been subtracted from the absorbance values obtained for all other TNFα concentrations.

The characterization of the microfluidic immunoassay based on the nitrocellulose substrate was first carried out with a simple chip containing only the main channel connected to an external syringe pump (Nemesys syringe pump, Cetoni GmbH) and a 1 mL glass syringe (Hamilton Company), so that an ideal control of the reagent flow rates was guaranteed. After all the incubation steps were performed as described in the protocol, the enzymatic products accumulated on the reaction area were displaced towards the absorbance measurement area at a flow rate of 3 µL/min. Figure 7a shows the corresponding photodiode signals for the cases of 0 and 10 ng/mL TNFα concentration in the test solution. The initial values correspond to the absorbance of non-oxidized TMB that has not been in contact with the nitrocellulose substrate during the last enzymatic reaction incubation. Then, a valley in the signal indicates that the blue oxidized TMB has reached the absorbance measurement area. Finally, transparent non-oxidized TMB fills this area again, and the photodiode signal returns to the initial values. This procedure was followed for various immunoassays with TNFα samples in the range of 0.6 to 20 ng/mL. The obtained dose-response curve is shown in Figure 7b. The absorbance was calculated using Equation (1) with V_ox_ being the minimum in the photodiode signal and V_non-ox_ the value recorded just after the incubation.

The limit of detection calculated from the measured data is 0.6 ng/mL. This is about one order of magnitude larger than typical values obtained for conventional ELISA assays [31]. This may be related to the much shorter incubation times used in the present microfluidic enzyme-linked immunoassay. Nevertheless, the presented immunoassay was selected to assess the fluidic performance of the chip. Future work will be focused on improving its sensitivity and reproducibility.

**Figure A4:**
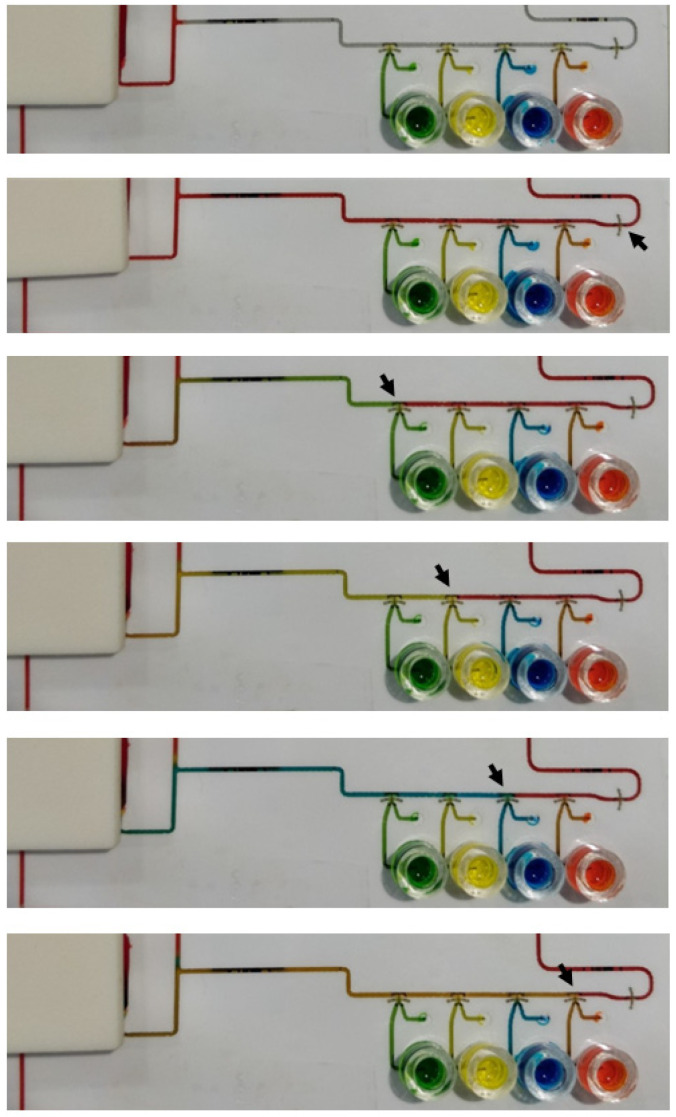
Images of the chip at different steps of an assay-like test. The black arrows indicate the open valves.

**Figure A5:**
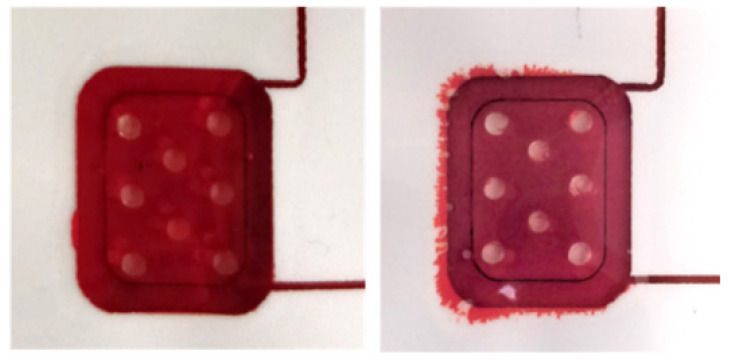
Images of the pump before and after an assay.

Finally, the chip for fully automated immunoassay including 6 wax valves and integrated pumping chamber was tested with a 10 ng/mL TNFα solution. Four assays were carried out. All the valves opened and closed as programmed, and the different steps of the immunoassay protocol proceeded as expected. However, the photodiode signal did not show any appreciable variation during the readout. Additional tests using different reagent dying for better visualization of their flow through the main channel were performed. Figure A4 and Video S1 show the results. Since the sample and reagents were flowing through the main channel in the same way as in the experiments carried out with the external syringe pump, the only step that was susceptible to be significantly different was the incubation of the TMB at stop-flow conditions. We investigated further if the integrated pump was capable of completely stopping the flow after the injection of the TMB in the channel, which was critical to allow the accumulation of enzymatic reaction products. To that end, in a new experiment, the remaining TMB solution was removed from the reservoir just at the beginning of the incubation period. In this way, we could visualize that air was slowly entering the channel during the incubation, which indicated that the flow had not been completely stopped. We associate this problem with a failure of the PSA adhesion along the perimeter of the pump chamber (Figure A5). A slow detachment of the layers can produce an increase in the chamber volume, which in turn can generate the observed residual flow. New designs and materials will be tested in the future to solve this limitation of the integrated pump.

## 4. Conclusions

A new microfluidic platform with LED light-actuated valves has been presented, and its potential for the automation of ELISA assays has been demonstrated. The valves have been characterized, and the platform’s working principle, based on the use of a photo-thermal heater to create and refill a tunnel in a wax barrier, has been elucidated. The integration of additional components with the lab-on-a-foil technology used, including reagent reservoirs, a pumping chamber, and a functionalized nitrocellulose solid phase for the target analyte capture, has also been demonstrated. The need to improve the pumping chamber design to achieve stop-flow conditions has also been identified. Future work on this microfluidic platform can also include a better characterization and control of the heater composition and photothermal properties, study of the wax-melting dynamics, and valve design improvements that can lead to the optimization of energy consumption and valve reliability.

Compared to other approaches using pneumatic or mechanical actuation, the use of LED light for actuating the valves enables a more compact instrument, even if large number of valves are required. The simple Arduino-controlled instrumentation developed here is capable of addressing up to 64 valves. The integration of a larger number of valves will pave the way for new applications and functionalities. Chips with multiple assays for multiplexed biomarker detection, which can also include blanks and controls for quantification, are envisioned.

## Supplementary Materials

The following supporting information can be downloaded at: https://www.mdpi.com/article/10.3390/bios12050280/s1, Video S1: Sequential opening and closing of two valves using dyed solutions.
